# Trachoma Decline and Widespread Use of Antimicrobial Drugs

**DOI:** 10.3201/eid1011.040476

**Published:** 2004-11

**Authors:** Jaya D. Chidambaram, Mariko Bird, Vivian Schiedler, Alicia M. Fry, Travis Porco, Ramesh C. Bhatta, Hem Jha, J.S.P. Chaudary, Bruce Gaynor, Elizabeth Yi, John P. Whitcher, Susie Osaki-Holm, Thomas M. Lietman

**Affiliations:** * University of California, San Francisco, California, USA;; † Centers for Disease Control and Prevention, Atlanta, Georgia, USA;; ‡ California Department of Health Services, Berkeley, California, USA;; § Geta Eye Hospital, Geta, Nepal

**Keywords:** trachoma, chlamydia, azithromycin, elimination, antibiotics, perspective

## Abstract

Trachoma is disappearing in many parts of the world, even in the absence of specific control programs. Following mass antimicrobial drug treatments for trachoma in western Nepal, the prevalence of trachoma declined far more rapidly than could be attributed to the control program alone. Pharmacy surveys in the same region found that children received more antichlamydial drugs from sources outside the trachoma program than they did from the program itself. We demonstrate that high background antimicrobial drug use may be responsible for much of the observed decline in trachoma and discuss its potential role in eliminating this infectious disease.

## Abstract 2

Widespread use of antimicrobial drugs may be contributing to trachoma decline.

Trachoma is disappearing in many parts of the world, even in the absence of specific control programs. It is a disease of the rural poor, and as living conditions have improved during the last century, a corresponding decline in trachoma has occurred (*1**–**4*). In Western Europe and the United States, trachoma virtually disappeared by the late 20th century. Other infectious diseases such as syphilis, chancroid, tuberculosis, and leprosy also began to subside in Europe and the United States during this time. This downward trend seems to have begun before, and continued into, the antimicrobial drug age. Therefore, many attribute this decline to socioeconomic factors, such as improved sanitation and social changes, and even to legislation to control venereal disease, rather than to antimicrobial drugs. Addressing the importance of antimicrobial agents in the disappearance of these infectious diseases retrospectively is difficult. In the case of trachoma, we have a unique opportunity to observe the effect of rising antimicrobial pressure in the community on a disease that is in decline but has not yet disappeared.

From 1998 to 2001, a region of western Nepal was monitored for trachoma prevalence, following mass antimicrobial drug distribution for trachoma. A dramatic fall in disease prevalence was observed that could not be attributed to the effect of the trachoma control program alone (*5*). We conducted a survey of pharmacies in the same region and found a surprisingly large quantity of antimicrobial drugs were being used for indications other than trachoma control (*6*). Here, we evaluate whether this background antimicrobial use may be responsible for the downward secular trend in the prevalence of trachoma.

## Analysis of Decline in Trachoma Prevalence in Western Nepal

From May 1998 to May 2001, a total of 25 villages from three subdistricts (known as Village Development Committees) in the Kailali and Konchapur districts of far-western Nepal were monitored for clinically active trachoma. During this time, an annual mass azithromycin treatment program began. At each visit, all children 1–10 years of age were examined for signs of clinically active trachoma by using the World Health Organization (*7*) simplified trachoma grading system (*8*). In total, >20,000 examinations were performed; 180–650 children were examined during each village visit (*5*). The presence of a secular trend, a downward trend independent of the trachoma program, was evaluated by monitoring one third of the villages for 6 months before any antimicrobial drug treatment was given. Seasonal variation was determined by performing village visits in both the spring and the fall. No other specific trachoma prevention activities such as hygiene, fly control, or water supply programs were instituted during the course of this study (*9*).

Trachoma prevalence data were analyzed by using a multivariate autoregression (AR1) model with the following covariates: effect of the trachoma program, seasonal variation, and secular trend. The analysis showed that the trachoma program's distributions of antimicrobial drugs alone could account for some, but not all, of the observed reduction in clinically active trachoma (*5*). A substantial proportion of the decrease in trachoma prevalence 6 months posttreatment was attributable to a secular trend, independent of the trachoma program's effect and seasonal changes (26% decrease, p < 0.001, 95% confidence interval [CI] 15%–35% decrease).

## Antimicrobial Pressure from Outside the Trachoma Program

From February to May 2000, all pharmacies and government health posts in the Geta subdistrict of Kailali were surveyed to establish the total quantity of antimicrobial drugs distributed. All of these will be called pharmacies for the purposes of this article. Information obtained included the number of years each medicine hall had been open and, for each patient, age, antimicrobial agent, amount distributed, and patient's village. Pharmacy purchase receipts from this time period were also collected for analysis. The survey was repeated in September 2001 to gain additional patient information and to ensure that no gross seasonal variations occurred (*6*).

We analyzed these data to determine what percentage of the total antimicrobial agents distributed had antichlamydial activity. Susceptibility testing suggested that trimethoprim-sulfonamide combinations, tetracycline, macrolides, chloramphenicol, and amoxicillin are all effective against chlamydia. Also, other penicillins, cephalosporins, and the fluoroquinolones (ciprofloxacin and norfloxacin) are less effective antichlamydial agents (*10*). However, susceptibility testing for chlamydia has been difficult to standardize (*11*), and alternative assumptions could alter these percentages somewhat. For example, including ciprofloxacin, which has some effect against *Chlamydia trachomatis*, would have increased the proportion effective against chlamydia by 12%, but we used the lower, more conservative figure for analysis. To facilitate direct comparison of different antimicrobial agents, the total amount of antichlamydial antimicrobial drugs was converted into the standardized unit of defined daily doses (DDD). DDD is defined as the assumed average maintenance dose per day for a drug used for its main indication in adults (*12*). For children, the number of prescriptions given per child per year was calculated with 1998 census data. Both DDDs and the prescriptions per person-year are convenient measures to compare antimicrobial pressure, although neither is ideal; DDDs do not take into account the duration of each drug's antichlamydial activity, and prescriptions are not for a uniform amount of medication.

**Figure 1:**
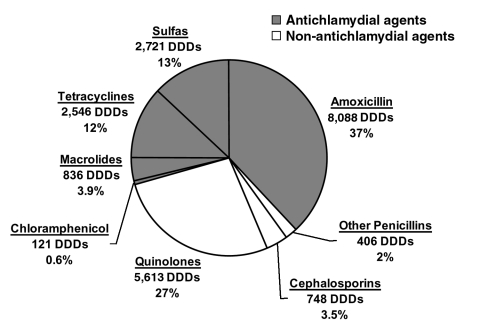
Antimicrobial drug use in Geta, Nepal. Antimicrobial drug sales in a 3-month period (mid-February to mid-May 2000) from all pharmacies in the Geta subdistrict, expressed as defined daily doses (DDDs) and as a percentage of the total DDDs sold (*6*). The shaded region represents antimicrobial drugs that are effective against *Chlamydia trachomatis*.

We estimated that pharmacies in Geta distributed 3.0 DDD of antimicrobial drugs per person per year in 2000 (Table). Sixty-eight percent of these prescriptions were effective against chlamydia (Figure 1). Thus, pharmacies distributed 2.0 DDD per person per year of antichlamydial agents. Forty-nine percent of all antimicrobial agents were distributed to children 0–10 years of age, and 33% to preschool children 0–5 years of age. We estimated that on average 1.2 prescriptions of antichlamydial agent are given to each preschool child per year (Table).

**Table:** Comparison of antimicrobial drug use within and outside a trachoma program^a^

	DDD/person/ year to all ages	Prescriptions/person/ year to preschool children
Antimicrobial pressure from pharmacies		
All antimicrobial drug prescriptions (from survey)	3.0	1.8
Antichlamydial prescriptions (from survey)	2.0	1.2
Antimicrobial pressure from trachoma program		
Annual azithromycin treatment with 100% coverage (theoretical)	2.3	1
Annual azithromycin treatment with 80% coverage (theoretical)	1.9	0.8

^a^Pharmacy survey data showing the total quantity of antimicrobial drugs used in Geta subdistrict and the proportion of antimicrobial drug with antichlamydial activity. An annual trachoma program theoretically gives 1 g of azithromycin to every adult (3.3 DDDs) and a lower dosage to children, averaging approximately 2.3 DDDs per person for all ages. We estimate from a mathematical model that mass antimicrobial treatment every 1.7 years would be sufficient to eventually eliminate ocular chlamydial infection from this region (*13*). DDD, defined daily dose, the average adult daily dosage for a drug's primary indication.

**Figure 2:**
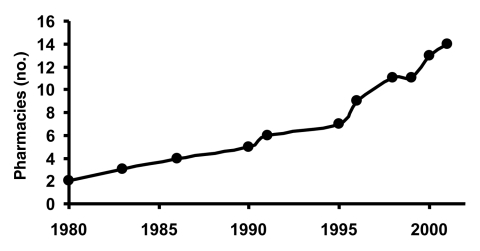
Number of pharmacies in Geta, Nepal. The number of pharmacies in Geta subdistrict increased from 2 in 1980 to 14 in 2001.

The number of pharmacies in Geta subdistrict has increased from 2 to 14 within the last 20 years, coinciding with the decrease in trachoma prevalence in the Tarai region of Nepal (Figure 2) (*2*). Eight of these pharmacies (57%) have been open for <5 years, and 10 (71%) for <10 years. In the last 20 years, the number of pharmacies has increased sevenfold, while the population of Geta has grown by approximately twofold, which suggests that more than three times as many pharmacies exist per person currently than in 1980.

## Antimicrobial Drug Use within the Trachoma Program

The trachoma control program in Kailali and Konchapur distributed single-dose oral azithromycin annually, as per World Health Organization (WHO) guidelines and covered an estimated 80% of the targeted population with its antimicrobial treatments (*5**,**9*). One gram of azithromycin is the recommended single dose in an adult to treat ocular chlamydial infection. This dose is equivalent to 3.3 DDD/person (*12*). For children, the recommended single dose of azithromycin is 20 mg/kg. The average dose for all ages (adults and children) was found to be approximately 2.3 DDD/person (*9*). With a treatment coverage of 80% of the entire population as recommended by WHO, a trachoma program would therefore administer 1.8 DDD/person at each mass distribution of antimicrobial agents.

## Antimicrobial Drug Use Necessary for Elimination of Trachoma in Western Nepal

Using a previously described mathematical model, we estimated the frequency of mass azithromycin distributions and the amount of antimicrobial drug needed to eliminate infection from this region of western Nepal (*13*). Before treatment, the average prevalence of active trachoma was 17% in children 1–10 years of age in western Nepal (*5*). With antimicrobial drug treatment that is 95% effective in a person and with 80% coverage of the population, the model indicates that mass treatments would be needed every 1.7 years (20.4 months) in western Nepal to progressively reduce the prevalence of active trachoma. Therefore, mass treatments given annually would be more than enough to eliminate ocular chlamydial infection.

## Discussion

The amount of antichlamydial drugs given out by pharmacies in Geta (2.0 DDD/person/year) is slightly more than the estimated amount that would bring about the elimination of ocular chlamydial infection in this region of western Nepal (1.9 DDD/person/year). Children, in particular preschool children, are by far the most likely to harbor ocular chlamydia. Pharmacies distributed nearly one half of the total antimicrobial agents to children 0–10 years of age, and one third to children 0–5 years of age. Preschool children received 1.2 prescriptions per year of antimicrobial drugs that are effective against chlamydia, which is far more than the estimated 0.6 per year that would eliminate infection. We therefore conclude that antibiotics given for reasons other than trachoma control may play a role in the disappearance of trachoma in this region.

The prevalence of active trachoma has decreased in many regions of the world in the absence of programs specifically targeting this disease (*1**–**4**,**14**,**15*). From 1981 to 1996, active trachoma in children declined from 30% to <10% in each of two adjacent districts of western Nepal; one district had an intense trachoma control program; the other district did not (*2*). Surveys in the Kailali and Konchapur districts of western Nepal have shown a large secular trend, suggesting that active trachoma would have disappeared rapidly even if a trachoma program had not been implemented (*5*). This situation is not unique to Nepal. A village in Gambia had hyperendemic trachoma in 1959 (66% prevalence in children), yet a followup survey in 1987 found that active disease had nearly disappeared, after only a modest 2-year control program of tetracycline administration (*1*). A study in Malawi showed a 50% reduction in active trachoma over a 16-year period in the absence of a specific trachoma program (*4*).

What might be the cause of this secular trend seen in so many countries? Various socioeconomic factors have been associated with the disappearance of trachoma, but studies have had difficulty establishing causality for any of them (*16**–**18*). In particular, facial hygiene and fly density are both believed to be related to trachoma activity (*19**–**21*). Several studies have associated dirty faces with active trachoma (*22**,**23*), but a trial involving intensive face-washing produced a modest (and statistically insignificant) decrease in clinically active trachoma at 1 year (*21*). The face fly (*Musca sorbens*) has been implicated as a vector of trachoma (*24**,**25*). A recent study in the Gambia found that regular insecticide spraying in villages did reduce active trachoma (*25*); however, future controlled studies are necessary to determine the sustainability of this promising measure.

What role have antimicrobial agents played in the disappearance of trachoma? In a person, ocular chlamydial infection can be successfully treated with a single dose of azithromycin (*26**,**27*). At the community level, controlled trials in Tanzania, Gambia, and Egypt have shown that a single course of azithromycin can markedly reduce ocular chlamydial infection, even 1 year later (*28*). Our findings in this study support the hypothesis that the rising use of antimicrobial drugs in the community for indications other than trachoma may contribute to the disappearance of this disease.

Several of the principal antimicrobial drugs used in Nepal for systemic infectious diseases have antichlamydial action. National treatment guidelines for childhood pneumonia recommend co-trimoxazole (a combined preparation of sulfonamide and trimethoprim) as the treatment of choice, followed by amoxicillin or oral chloramphenicol as second-line therapy (*29*). Other childhood infectious diseases are treated according to the adapted WHO Integrated Management of Childhood Illness (*30*). WHO recommends chloroquine as the first-line therapy for malaria in Nepal, and sulfadoxine-pyrimethamine for chloroquine-resistant cases (*31*). The latter drug has antichlamydial activity through its sulfonamide component, sulfadoxine.

Why trachoma is disappearing should be investigated before it is gone, so that this knowledge can be applied to other diseases. If infection in a region is already in decline, the effect attributed to a trachoma control program may be exaggerated, and the program's success may not be duplicated in less fortunate areas. Conversely, beneficial factors could be introduced in areas where a downward secular trend does not already exist. Much discussion has taken place about the dangers associated with the indiscriminate use of antimicrobial drugs. These problems should be balanced against the benefits. The widespread use of antimicrobial drugs in developing countries for indications other than trachoma may play a role in eradicating one of the world's leading causes of preventable blindness.
